# Sex, Diet, and the Social Environment: Factors Influencing Hair Cortisol Concentration in Free-Ranging Black Bears (*Ursus americanus*)

**DOI:** 10.1371/journal.pone.0141489

**Published:** 2015-11-03

**Authors:** Diana J. R. Lafferty, Mark L. Laudenslager, Garth Mowat, Doug Heard, Jerrold L. Belant

**Affiliations:** 1 Department of Wildlife, Fisheries and Aquaculture, Carnivore Ecology Laboratory, Mississippi State University, Mississippi State, Mississippi, United States of America; 2 Department of Psychiatry, Behavioural Immunology and Endocrinology Laboratory, University of Colorado Denver, Anschutz Medical Campus, Aurora, Colorado, United States of America; 3 British Columbia Ministry of Forests, Lands, and Natural Resource Operations, Nelson, British Columbia, Canada; 4 British Columbia Ministry of Forests, Lands, and Natural Resource Operations, Prince George, British Columbia, Canada; 5 Department of Forestry and Environmental Resources, Fisheries, Wildlife and Conservation Biology Program, North Carolina State University, Raleigh, North Carolina, United States of America; University of Tasmania, AUSTRALIA

## Abstract

Increasingly, measures of glucocorticoid levels (e.g., cortisol), key components of the neuroendocrine stress axis, are being used to measure past hypothalamic-pituitary-adrenal (HPA) activity to index psychological and physiological stress exhibited by wildlife for assessing individual and population-level well-being. However, many intrinsic and extrinsic factors affect HPA activity in animals. Using American black bears (*Ursus americanus*; *n* = 116) as an ecological model and hair cortisol concentration (HCC) as an integrative measure of past HPA activity, we evaluated the influence of diet, sex and the social environment on black bear HCC in a free-ranging population that spanned adjoining ecoregions with differing densities of potential conspecific and heterospecific competitors. HCC varied by sex, with female HCC ranging from 0.6 to 10.7 pg/mg (median = 4.5 ± 1.2 mean absolute deviation [MAD]) and male HCC ranging from 0.5 to 35.1 pg/mg (median = 6.2 ± 2.6 MAD). We also observed a three-way interaction among sex, δ^14^C and ecoregion, which may indicate that some differences in HCC between female and male black bears results from variability in the nutritional needs of larger-bodied males relative to smaller-bodied females, slight differences in food resources use between ecoregions as well as sex-based differences regarding the social environment. Once we understand what drives sex-specific differences in HCC, HCC may aid our understanding of the physiological responses by bears and other wildlife to diverse environmental challenges.

## Introduction

Understanding the physiological response of wildlife to their environment is fundamental to evolutionary biology, ecology and conservation. Arguably, one of the most important physiological responses by wildlife to environmental stimuli is activation of the hypothalamic-pituitary-adrenal (HPA) axis, which results in the release of stress hormones such as glucocorticoids (e.g., cortisol) [1]. Activation of the HPA axis may occur in response to environmental challenges such as resource competition or in anticipation of seasonal environmental changes resulting in increased energetic demands (e.g., hyperphagia, reproduction, migration) [2]. While short-term elevated cortisol levels can trigger adaptive behavioral responses that aid in escape or defense [3, 4] or facilitate shifts in life-history strategies [3, 5], chronic activation of the HPA axis can hinder vital function leading to suppressed immune function, muscle wasting, weight loss, and the reduction or absence of reproduction [6–9].

Increasingly, measures of cortisol are used to quantify past HPA activity to index psycho-physiological stress experienced by wildlife [1, 2, 10]. Cortisol and cortisol metabolites can be assayed from multiple biological matrices including blood, urine and feces [1, 10, 11]. Hair cortisol concentration (HCC), however, provides an integrative measure of past HPA activity over the growth period of the hair (e.g., weeks to months) [1, 12, 13] much like hemoglobin A1c corresponds to glucose control over the past three months [14]. As such, hair provides a matrix in which to measure long-term stress rather than acute stress or hormonal fluctuations influenced by circadian rhythms [13].

However, myriad intrinsic and extrinsic factors as well as predictable and unpredictable environmental changes can influence stress responses in wildlife. For example, age, sex, social status and past experience can influence the physiological response of animals to environmental challenges [2, 4, 15]. In addition, the abundance and quality of food [6, 16], the social competitive environment within and between species [17], extreme weather events [18] and anthropogenic disturbances [11] can influence HPA activity. Moreover, wildlife capture and handling for research purposes has been shown to influence HCC [19]. These factors also may interact, confounding our ability to interpret HCC as a proxy to stress in wildlife [2]. Thus, non-invasive methods may be better suited for examining HCC patterns in free-ranging wildlife populations and for advancing our knowledge about the ecological and evolutionary importance of intrapopulation variation in physiological responses by wildlife to diverse environmental situations.

Using American black bears (*Ursus americanus*) as model species, and using non-invasive sampling methods, we tested multiple hypotheses regarding the influence of diet, sex and the social environment on HCC in a free-ranging population that spanned adjoining ecoregions with differing absolute and relative black bear and grizzly bear (*Ursus arctos*) densities. First we hypothesized that if a link exists between diet and HPA activity, then differences in HCC would be associated with dietary niche differences. For example, individuals or population segments (e.g., sex-class) foraging at a higher trophic level (e.g., eating animal matter) as indicated by the results of diet estimation based on stable isotope analysis, should have lower HCC because consumption of high-quality resources should confer nutritional benefits resulting in lower nutritional stress [17]. Alternatively, if females and males have similar dietary niches, then males may exhibit higher overall HCC if the physiological constraints of maintaining a larger body size results in nutritional stress. However, if nutritional requirements are met across the range of dietary niches observed, then we would expect no differences in HCC associated with dietary niche differences.

Second, we hypothesized that female black bears, which are competitively subordinate to males, would exhibit higher HCC and greater among-individual variation in HCC due to differences in reproductive states (i.e., with or without dependent young), which can influence female foraging behavior and social interactions [20] as well as physiological condition [21]. Although the age structure of the population is unknown, age at first reproduction in females can vary from two to six years of age [22–24] and females may not rear cubs-of-the-year in consecutive years [22]. Alternatively, our third hypothesis was that if the social competitive environment was an important driver of HCC in black bears, then differences in HCC would be associated with differences in black bear and grizzly bear densities across the study area, which would be most pronounced in male black bears. Specifically, male dominance hierarchies associated with breeding as well as potential variability in interactions with grizzly bears could result in higher and more variable HCC among males. For example, if interspecific interactions with grizzly bears influences black bear HCC, we would expect male black bears in an ecoregion with higher grizzly bear density to have higher and more variable HCC. However, if intraspecific dominance hierarchies have a greater influences on black bear HCC, we would expect higher and more variable HCC in an ecoregion with higher black bear densities.

## Materials and Methods

### Ethics statement

Archived hair samples were provided by the British Columbia Ministry of the Forest, Lands and Natural Resource Operations and were originally obtained during provincially sponsored ursid inventory projects using methods approved by the British Columbia Provincial Animal Care Committee.

### Study area

The study area (54°39’N, 122°36’W) spanned two adjoining ecoregions in central-eastern British Columbia, Canada: Parsnip Plateau (hereafter, plateau: 3,016 km^2^) and Hart Ranges of the Rocky Mountains (hereafter, mountains: 6,436 km^2^). Ecoregions varied in black bear and grizzly bear densities (mountains: 100 black/49 grizzly/1000 km^2^; plateau: 257 black/17 grizzly/1000 km^2^) and in the extent of anthropogenic disturbances [25]. For example, the plateau was subjected to industrial development, extensive transportation infrastructure including a major highway, human settlements, and widespread logging over the previous several decades. Anthropogenic disturbance in the mountain ecoregion was less pervasive because logging operations were restricted to lower elevations and there were no permanent human settlements [26]. In addition, both ecoregions had similar relative abundances of terrestrial prey (D. Heard, Ministry of the Environment, BC, pers. obs.), whereas Chinook salmon (*Oncorhynchus tshawytscha*) were only available in a small portion of the mountain ecoregion.

### Sample collection and selection

We used an archived collection of black bear hair samples collected non-invasively during 30 May–2 August 2000 that were subjected previously to DNA analysis for species, sex and individual identification [25]. Briefly, barbed-wire hair collection stations were spaced systematically using a 16 km^2^ grid overlain across the study area as described by [27]. Hair collection stations consisted of a single strand of barbed-wire placed 50 cm above ground around a minimum of three trees (23). Decaying logs, branches and adjacent vegetation were piled in the center of the barbed-wire corral and a scent lure was applied to the debris pile as an attractant (23). From this archived black bear hair collection, we selected 32 female and 32 male bears from each ecoregion (*n* = 128). Because hair provides an integrative record of diet and HPA activity over the growth period of the hair, and because we selected mature guard hairs for analysis, we assumed hair samples represented diet and hormones assimilated the previous year (i.e., 1999) during the hair growth period [16, 17, 19, 28, 29].

#### Stable isotope analysis

To estimate diet, hair samples were analyzed for stable carbon and nitrogen isotope ratios at the Great Lakes Institute of Environmental Research (University of Windsor, Windsor, Ontario, Canada). One sample from each genetically identified individual was selected. Follicles were removed from whole guard hairs and the hair washed in a chloroform:methanol (2:1) solution using a sonicator bath at 30 degrees for 20 minutes, rinsed twice with distilled water, washed again with distilled water in a sonicator bath for 20 minutes and dried in an oven at 40 degrees Celsius for 24 hours. Whole hair samples were weighed, measured and analyzed for carbon (^13^C/^12^C) and nitrogen (^15^N/^14^N) stable isotope ratios using an Elemental Analyzer-Isotope Ratio Mass Spectrometer (EA-IRMS). We report isotopic signatures in delta (δ) notation such that δ^13^C or δ^15^N = [(Rsample/Rstandard)– 1] x 1000, where Rsample and Rstandard are the ^13^C/^12^C or ^15^N/^14^N ratios of the sample and standard, respectively. The standards are PeeDee Belemnite limestone for carbon and atmospheric N_2_ for nitrogen [30]. Analysis of internal laboratory standards suggested precision of 0.08‰ and 0.17‰ for δ^13^C and δ^15^N, respectively and National Institute of Standards and Technology (NIST) standards suggested an analytical accuracy of the instruments of 0.06‰ and 0.13‰ for δ^13^C and δ^15^N, respectively.

#### Cortisol extraction and assay validation

Cortisol analysis and assay validation were conducted at University of Colorado Denver Anschutz Medical Campus (Aurora, Colorado, USA) as previously described [31, 32]. Briefly, each hair sample was placed in a pre-weighed 2 ml cryovial (Wheaton, Millville, NJ, USA), washed three times in 100% isopropanol and dried. After washing, drying and re-weighing samples on a high sensitivity electronic balance (Mettler Toledo Model MS105, Greifense, Switzerland) to determine individual hair mass in these small samples, hair was ground in the same cryovial using a ball mill (Retsch, Haan, Germany) after adding a 4.76 mm carefully cleaned stainless steel ball bearing. Specially milled aluminum cassettes were designed to hold three cryovials. The cassettes, containing the cryovials, were submerged in liquid nitrogen for 3 to 6 minutes to freeze hair samples to facilitate grinding. Samples subsequently were ground for 4 to 5 minutes. Powdered hair was extracted in the same cryovial at a ratio of 5 mg hair/100 microliters high pressure liquid chromatography (HPLC) grade methanol for 24 hours at room temperature on a side-to-side shaker platform. Confining hair to the same cryovial during these initial steps allowed for working with smaller samples (e.g., lower weights) as there was no loss of hair during aforementioned weighing and grinding steps. Following methanol extraction, cryovials were spun for 10 minutes in a centrifuge at 1500 g to pellet the hair. The extraction supernatant was removed and placed into a microcentrifuge tube and dried under a stream of nitrogen in a drying rack in a fume hood at room temperature. The dried extracts were then reconstituted with assay diluent based on hair weight and methanol added. Cortisol levels were determined using a commercial high sensitivity Enzyme Immunoassay (EIA) kit (Salimetrics LLC, State College, PA, USA) per manufacturer’s protocol [31]. Methods for assay cross validation with other laboratories using liquid chromatograph-mass spectrometry (LC/MS) were described previously by Russell et al. [12]. Cross validation entailed assaying 10 identical samples by four laboratories by EIA and/or LC/MS and comparing the resulting levels. Correlations across laboratories of r^2^>0.9 were noted for both EIA and LC/MS indicating excellent consistency and comparability across laboratories [12].

Assay recovery was evaluated by spiking a black bear hair sample with the equivalent of 150 pg/mg and serially diluting the spike for comparison to the assay standard curve. Simple linear regression was used to assess spiking recovery between serially diluted hair extracts and cortisol standards in the same assay. Visual inspection and regressions suggested high recovery for diluted extracts of black bear hair [33] (Figure A in S1 File). Cortisol spiking recovery was 96.01% ± 0.06 SD based on five determinations of recovery of spikes that were serially diluted in extracts of black bear hair (Figure B in S1 File). High and low quality commercial controls provided with each kit ran within expected ranges. As an additional control, human hair samples (i.e., internal laboratory control samples) were included to assess variability across assays due to variations in immunoassay procedures or the extractions. The control human hair was extracted for each assay as described above to quantify intra-assay (1.9%) and inter-assay (11.6%) coefficients of variation. Assay sensitivities for hair weights of 5, 10, and 20 mg are 0.31, 0.16, and 0.08 (pg/mg) respectively. Cross-reactivity for cortisol was provided by the manufacturer (Table B in S1 File). Overall this assay approach has wide acceptance for assessing cortisol in a variety of species including bears with excellent validation characteristics [10, 31, 34–38].

### Assessing intraspecific dietary niche variation

To assess intraspecific dietary niche variation between sexes and ecoregions, we used a multivariate Bayesian ellipse technique (Stable Isotope Bayesian Ellipses in R [SIBER]; [39]). Standard ellipse areas (SEA) and small sample size corrected ellipses (SEAc) were estimated using approximately 40% of the bivariate isotope data that best explained covariance, and by an error term associated with each ellipse that was generated by resampling the bivariate data 10^6^ times [39]. From the proportional outcome of repeated sampling, we generated 95% Bayesian credible intervals (CI) for each SEA_C_, enabling us to compare SEAc sizes between sexes within and between ecoregions [39].

We used general linear models to evaluate effects of sex, diet (δ^13^C and δ^15^N) and ecoregion on black bear HCC. Ecoregion and sex were assigned as factor variables, δ^13^C and δ^15^N were included as continuous covariates and were not correlated (*r* = 0.03), and hair mass (mg) was included as a continuous covariate to account for variation in assay sensitivity due to sample weight when extracting cortisol from hair of variable weight. We scaled δ^13^C, δ^15^N, and hair mass and regression assumptions were met by natural-log (ln) transforming the response variable, HCC. We ranked models using Akaike’s Information Criterion with small sample correction (AICc) and considered models competing if ≤ 2 AICc from the top model [40].

## Results

**Fig 1:**
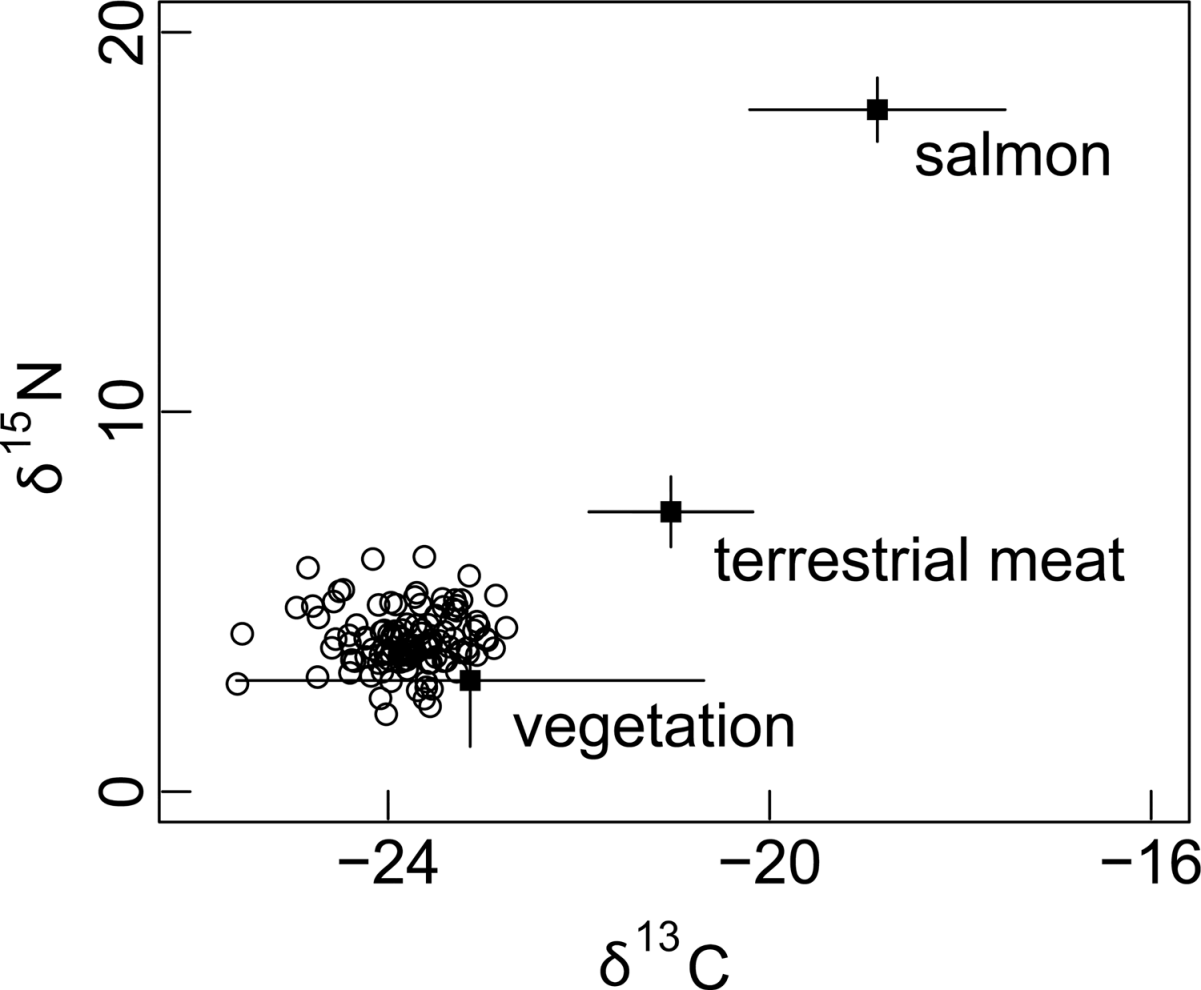
Distribution of black bears (*Ursus americanus*) in isotopic space (δ^13^C and δ^15^N) relative to mean food source values. Trophic discrimination factors were applied to each food category, which are represented by the mean δ^13^C and δ^15^N (± SD) of each food category. Black bears sampled from Parsnip Plateau and Hart Ranges of the Rocky Mountains, British Columbia, Canada, 1999.

We obtained stable isotope values and HCC from 116 individuals (29 female and 28 males from the mountain ecoregion, and 29 females and 30 males from the plateau ecoregion). Stable δ^13^C and δ^15^N values ranged from –25.6 to –22.8 (2.8‰) and 2.0 to 6.2 (4.2‰), respectively (Fig 1). The narrow range of black bear stable isotope values relative to generalized stable isotope values representing three potential major dietary components reflected a predominately herbivorous diet (Fig 1).

**Fig 2:**
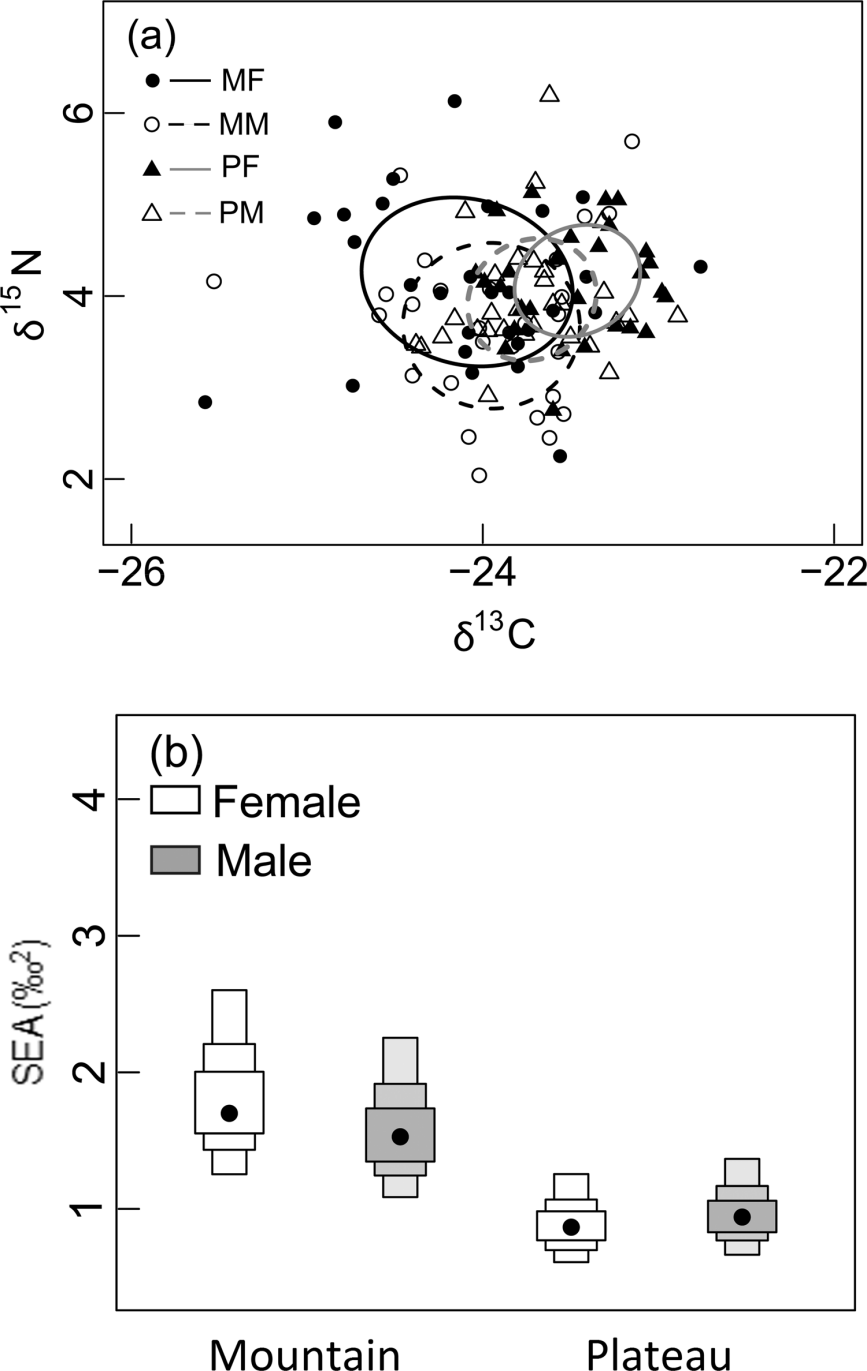
Black bear (*Ursus americanus*) isotopic niches and corresponding isotopic niche density plots. (a) Standard ellipse areas corrected for small sample size (SEAc), representing core (40%) dietary niches of black bear females (MF) and males (MM) from the mountain ecoregion and females (PF) and males (PM) from the plateau ecoregion. (b) Density plot representing the posterior probability distribution of SEAc sizes. Black dots correspond to means and decreasing bar widths represent 50%, 75% and 95% Bayesian credible intervals. Black bears sampled from Parsnip Plateau and Hart Ranges of the Rocky Mountains, British Columbia, Canada, 1999.

Although we found intrapopulation dietary niche differences, core dietary niches overlapped between sexes within and between ecoregions (Fig 2A). Specifically, the size of the dietary niche of females from the mountain ecoregion (1.73‰^2^) was greater than the size of the dietary niche of females (0.68‰^2^; *p* = 0.008) and males (0.76‰^2^; *p* = 0.003) from the plateau ecoregion (Fig 2B). Similarly, the size of the dietary niche of males from the mountain ecoregion (1.42‰^2^) was greater than the size of the dietary niche of females (*p* = 0.020) and males (*p* = 0.016) from the plateau (Fig 2B) ecoregion. However, sizes of dietary niches did not differ between sexes within the mountain (*p* = 0.709) or plateau (*p* = 0.630) ecoregions (Fig 2B).

**Fig 3:**
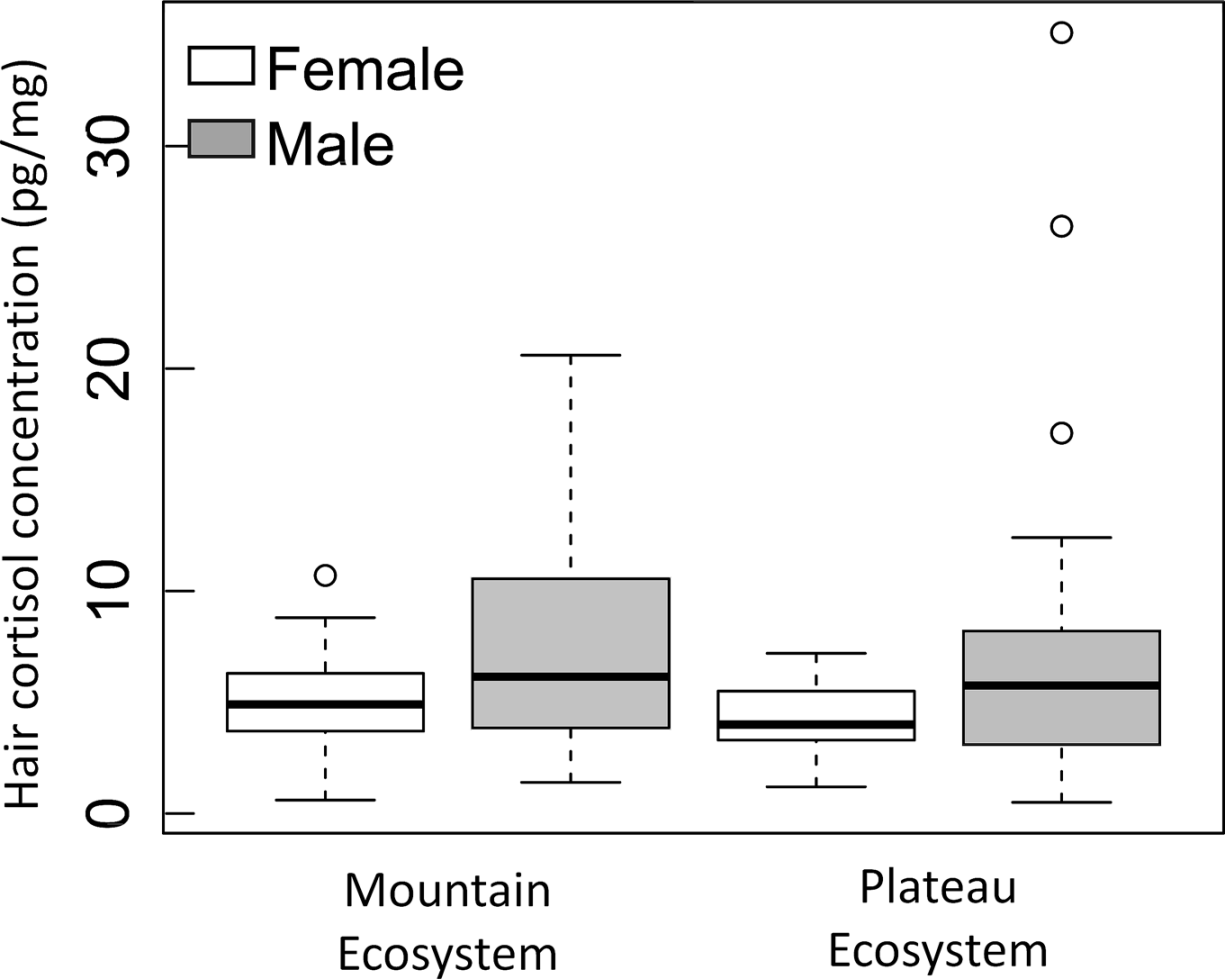
Black bear (*Ursus americanus*) hair cortisol concentration by sex and ecoregion. Black bears sampled from Parsnip Plateau and Hart Ranges of the Rocky Mountains, British Columbia, Canada, 1999.

We had 16 competing models explaining observed HCC variation (Table 1) and we used model averaging to examine coefficients using 95% confidence limits (Table 2). HCC varied by sex (Fig 3) with males having, on average, 62% greater HCC than females, although sex had low explanatory power (adjusted R^*2*^ = 0.06, β = 0.34, SE = 0.13, CI 0.08–0.61 [ln-transformed data]; Table 2). Among females, HCC ranged from 0.6 to 10.7 pg/mg (untransformed median = 4.5 ± 1.2 mean absolute deviation [MAD]), whereas variation was greater among males (untransformed range = 0.5–35.1 pg/mg; median = 6.2 ± 2.6 MAD). However, among-individual HCC variation in females did not differ between the mountain and plateau ecoregions, nor did among-individual HCC variation in males differ between ecoregions. We also found a three-way interaction among sex, δ^13^C and ecoregion that had low explanatory power (adjusted R^*2*^ = 0.10, β = 0.60, SE = 0.30, CI 0.02–1.19 [ln-transformed data]; Table B in S1 File). For example, black bears inhabiting the plateau ecoregion had slightly enriched carbon values relative to black bears from the mountain ecoregion, whereas the diet of black bears in the mountains spanned a wider range of carbon values and overall, male black bears had higher and more variable HCC values than females. Although hair mass was highly variable (range = 1.20–43.59 mg; median = 14.19), variation in hair mass did not help explain observed HCC variation as indicated by the confidence interval broadly overlapping zero (β = -0.06, SE = 0.07, CI = -0.20–0.06; Table 2).

**Table 1:** Linear models for explaining black bear (*Ursus americanus*) hair cortisol concentration.

Model^a^	k ^b^	ΔAIC^c^	LL^d^	Wt ^e^	Adj. *R* ^2^ ^f^
sex	3	0.00	-114.54	0.11	0.06
sex + δ^13^C	4	0.03	-113.48	0.10	0.07
sex + δ^13^C × ecoregion	6	0.31	-111.42	0.09	0.08
sex + δ^13^C + δ^15^N	5	0.87	-112.81	0.07	0.07
sex + δ^15^N	4	0.90	-113.92	0.07	0.06
sex + ecoregion	4	0.98	-113.96	0.07	0.06
sex × δ^13^C	5	1.09	-112.92	0.06	0.07
sex + δ^13^C + hair mass	5	1.16	-112.96	0.06	0.07
sex + hair mass	4	1.35	-114.14	0.05	0.05
sex + δ^13^C × ecoregion + δ^15^N	7	1.41	-110.83	0.05	0.08
sex × δ ^13^C + δ^13^C × ecoregion	7	1.60	-110.93	0.05	0.08
sex + δ^13^C × ecoregion + hair mass	7	1.67	-110.97	0.05	0.08
sex + ecoregion + δ^15^N	5	1.68	-113.22	0.05	0.06
sex × δ^13^C + hair mass	6	1.72	-112.12	0.05	0.07
sex × δ^13^C × ecoregion	9	1.87	-108.73	0.04	0.10
sex + δ^13^C + ecoregion	5	1.95	-113.35	0.04	0.06
null	1	5.62	-118.40	0.00	0.00

Black bears sampled from Parsnip Plateau and Hart Ranges of the Rocky Mountains, British Columbia, Canada, 1999.

^a^ Models with interaction terms also include main effects.

^b^ Number of model parameters.

^c^ All competing models are shown and are ranked in ascending order by Akaike’s information criterion (AIC) adjusted for small sample size.

^d^ Maximum log likelihood.

^e^ Model weight.

^f^ Measure of model fit for each model.

**Table 2:** Model averaged coefficients for parameters in competitive models (ΔAICc < 2 from top model) explaining cortisol levels in black bears (*Ursus americanus*). Data were natural-log transformed.

			95% confidence limits	
Parameter	Estimate	SE	Lower	Upper
Intercept	1.44	0.10	1.23	1.64
δ^13^C	-0.13	0.09	-0.30	0.04
sex (male)^a^	0.34	0.13	0.08	0.61
ecoregion (plateau)^b^	-0.10	0.15	-0.40	0.19
δ^13^C × ecoregion (plateau)^b^	0.23	0.19	-0.14	0.61
δ^15^N	0.07	0.06	-0.05	0.19
sex (male)^a^ × δ^13^C	0.09	0.16	-0.22	0.41
hair mass	-0.06	0.07	-0.20	0.06
sex (male)^a^ × ecoregion (plateau)^b^	-0.12	0.28	-0.67	0.43
sex × δ^13^C × ecoregion (plateau)^b^	0.60	0.30	0.02	1.19

Black bears sampled from Parsnip Plateau and Hart Ranges of the Rocky Mountains, British Columbia, Canada, 1999.

^a^ Female is the reference group.

^b^ Mountain ecoregion is the reference group.

## Discussion

Our hypotheses that female black bears would exhibit higher HCC and greater among-individual variability in HCC than males as a consequence of subordinate social rank and variation in reproductive states (e.g., with cubs, without cubs) was not supported. In fact, at the population-level, male black bears generally exhibited higher HCC than females as well as greater among-individual HCC variation. Although recent studies that measured fecal cortisol metabolites in diverse mammalian taxa, including red squirrel (*Tamiasciurus hudsonicus*) [41] and African lions (*Panthera leo*) [11] found that males of a species may have higher fecal cortisol metabolite levels than females, Bryan et al. [16] found no significant differences in cortisol between sexes in black bears or grizzly bears, whereas Cattet et al. [19] found higher HCC in female brown bears than male brown bears. Conflicting results among studies suggest that multiple factors may contribute to observed sex-based difference in stress hormone levels [2, 4, 41, 42].

As alternative hypotheses, we posited that male black bears may exhibit higher HCC and greater among-individual variation in HCC due to the social competitive environment associated with differences in black bear and grizzly bear densities across the study area. Yet despite marked differences in black bear and grizzly bear densities between ecoregions, we found no differences in the mean or variance of HCC between males from the two ecoregions or between females from the two ecoregion, and we therefore rejected this hypothesis. If interspecific social interactions with brown bears was an important factor in black bear HCC [16], we expected to find higher HCC and greater among-individual HCC variation among males in the mountain ecoregion where grizzly bear density was much higher and where there were fewer trees to provide escape cover for black bears. Similarly, if intraspecific dominance hierarchies were influencing HCC and driving greater among-individual HCC variation among black bears due to agonistic behavior between males associated with breeding, then we expected to find higher and more variable HCC among black bears on the plateau where black bear density was quite high [25].

As previously noted, recent studies have suggested that numerous factors may contribute to sex-based differences in stress hormone levels [2, 4, 19, 41, 42]. For example, male black bears may be more sensitive to human disturbances compared to females. Results from a meta-analysis comprising four vertebrate classes (i.e., Amphibia, Aves, Mammalia, Reptilia), suggested that males of a species, in general, may be more sensitive overall to human activities than females, regardless of disturbance type [2]. As both mountain and plateau ecoregions were subject to human disturbances during the sampling period, males may have been disproportionally affected, and thus mounted a greater hypothalamic pituitary adrenal axis response as reflected in slightly greater hair cortisol levels than females. It is also possible that differences in the amount of anthropogenic disturbance and grizzly bear densities were confounded, which limited our ability to detect whether either of these variables influence HCC. Specifically, there was extensive anthropogenic disturbance in the plateau ecoregion but relatively low grizzly bear density, whereas there was substantially less human activity in the mountain ecoregion but relatively high grizzly bear density, which may have resulted in black bears of both sexes mounting similar stress responses in both ecoregions.

In addition, the composition and size of the dietary niches of black bears differed between ecoregions, likely a consequence of a greater range in plant δ^13^C values along elevation gradients in the mountain ecoregion compared to the plateau [43]. However, diet and the sizes of dietary niches of females and males within ecoregions did not differ, yet males exhibited higher HCC than females, which may reflect nutritional stress associated with the physiological constraints of maintaining a larger body size relative to females on a predominantly plant-based diet [44, 45]. As such, we were unable to reject our hypothesis that diet influences black bear HCC.

Our results, particularly the finding of an interaction effect among sex, δ^13^C and ecoregion, support previous assertions that observed HCC differences between sexes are likely a consequence of multiple interacting factors. For example, Bryan et al. [16] found no evidence that grizzly bear density influenced black bear HCC directly, although HCC in black bears was affected by salmon availability, which was thought to mediate food resource competition between black bears and grizzly bears. Belant et al. [46] found that grizzly bears displaced female black bears from high-quality habitats containing salmon spawning streams and suggested that where black bears are sympatric with grizzly bears, black bears can reduce interspecific competition and meet their nutritional requirements by consuming a diet dominated by vegetation. Lafferty et al. [47] subsequently found that black bears in the Denali region of Alaska achieved similar percentage body fat, which is perhaps a higher-order index of fitness than HCC [48, 49], across the range of food resources consumed, indicating that black bears that co-occur with grizzly bears can achieve their nutritional needs on a predominantly herbivorous diet. As such, higher and more variable HCC in male black bears is likely a result of not only variation in the nutritional needs of individuals but also their social environment. However, we note that all models tested had low explanatory power and that factors other than those tested, such as social dominance rank or reproductive status, are likely of importance to physiological stress in black bears.

We recognize that a limitation of our study was the absence of behavioral observations regarding social dominance hierarchies within the population that are likely driven by differences in sex-age class and body size [50]. Recent studies have demonstrated a link between social rank and stress hormone levels with basal cortisol and cortisol metabolite levels in social mammals including non-human primates, carnivores, and ungulates often highest in dominant individuals [51–55], but see [56]. Because black bears exhibit social dominance hierarchies and adult males are the dominant social class within a population [50], male black bears may have had higher and more variable HCC due to social dominance relationships that could not be assessed with our data.

The study of HCC for applications in wildlife health and conservation physiology is in its infancy with existing methods requiring additional testing [1, 2, 10, 19]. For instance, we excluded 12 hair samples from statistical analyses because no cortisol was detected. Further, in regards to ursids, the temporal relationship between serum total cortisol concentration and HCC is ambiguous [19], thereby complicating interpretations about the biological relevance and temporal frame over which HCC represents [2]. Hair collected from different parts of the body also may have different cortisol concentrations [19]. Because we used samples obtained through noninvasive methods [25, 26], we acknowledge that hair samples used in our study likely came from several different parts of the body, which may have affected our results; although we observed little differences among individuals of the same sex. Despite these challenges, mounting evidence suggests that as a retrospective biomarker of endocrine activity in wildlife, including assessments from four species of bears [13, 16, 17, 19, 42, 48, 57, 58], measures of HCC may provide meaningful insight into the long-term physiological responses of individuals to their environment. Greater understanding of observed HCC patterns in non-invasively sampled, free-ranging wildlife can help build our understanding of the eco-evolutionary significance of intrapopulation differences in HCC, which also can inform conservation and management planning. However, for HCC to be a useful tool, factors that contribute to intrapopulation variability in HCC must be identified. As such, in addition to measures of diet, sex and densities of conspecific and heterospecific competitors, future studies would benefit from incorporating measures of reproductive condition (e.g., testosterone, estradiol), identifying presence of young, age data and when possible, social rank. Moreover, studies that evaluate linkages between HCC and measures of fitness (e.g., survival, reproduction) in wildlife would enhance the utility of HCC as a conservation tool. Macbeth et al. [48] found preliminary evidence that HCC in polar bears (*U*. *maritimus*) was inversely related to measures of growth (i.e., length, mass, body condition index [BCI; [59]] and previous research provides evidence of a direct relationship between growth and fitness in polar bears [60–62]. However, few studies have linked HCC in mammals to measures of fitness. Once we understand the drivers of intrapopulation differences in HCC and how HCC is related to measures of fitness, HCC will have tremendous potential to inform our understanding of the physiological stress burden experienced by wildlife due to diverse environmental challenges and inform our understanding of the eco-evolutionary consequences of that stress burden to individual and population-level well-being.

## Supporting Information

S1 File Supporting information. Generalized δ^13^C and δ^15^N isotopic values ± SD and discrimination factors (Δδ) ± SD used to examine the black bear (*Ursus americanus*) isotopic values (**Table A**). Cross-reactivity for antibodies used in black bear (*Ursus americanus*) hair cortisol assay (**Table B**). All data used to evaluate the influence of diet, sex, and social environment on black bear (*Ursus americanus*) hair cortisol concentration (**Table C**). Relationship between serially diluted extracted black bear (*Ursus americanus*) hair and cortisol spike (**Figure A**). Black bear (*Ursus americanus*) cortisol extraction efficiency based on five determinations of recovery (**Figure B**). Three-way interaction among carbon, sex, and ecoregion associated with hair cortisol concentration in black bears (*Ursus americanus*) (**Figure C**).
(DOCX)
