# Supplementary material for: Sex, Diet, and the Social Environment: Factors Influencing Hair Cortisol Concentration in Free-Ranging Black Bears (Ursus americanus)
Source: PLoS One. 2015 Nov 3;10(11):e0141489. doi: 10.1371/journal.pone.0141489 (PMC4631324; doi:10.1371/journal.pone.0141489)
Supplement: S1 File — Generalized δ13C and δ15N isotopic values ± SD and discrimination factors (Δδ) ± SD used to examine the black bear (Ursus americanus) isotopic values (Table A). Cross-reactivity for antibodies used in black bear (Ursus americanus) hair cortisol assay (Table B). All data used to evaluate the influence of diet, sex, and social environment on black bear (Ursus americanus) hair cortisol concentration (Table C). Relationship between serially diluted extracted black bear (Ursus americanus) hair and cortisol spike (Figure A). Black bear (Ursus americanus) cortisol extraction efficiency based on five determinations of recovery (Figure B). Three-way interaction among carbon, sex, and ecoregion associated with hair cortisol concentration in black bears (Ursus americanus) (Figure C). (DOCX) [file pone.0141489.s001.docx]

## Supporting Information

**Table A in S1 File. Generalized δ^13^C and δ^15^N isotopic values ± SD and discrimination factors (Δδ) ± SD used to examine the black bear (*Ursus americanus*) isotopic values.**

| Food category | δ^13^C (‰) ± SD | Δδ^13^C_tissue-diet_ (‰) ± SD | δ^15^N (‰) ± SD | Δδ^15^N_tissue-diet_ (‰) ± SD |
| --- | --- | --- | --- | --- |
| Plant^a^ | -29.23 ± 2.44^a^ | 6.09 ± 1.42^d^ | -2.67 ± 1.74 | 5.60 ± 0.21^d^ |
| Terrestrial meat^b^ | -24.12 ± 0.87^b^ | 3.26 ± 0.51^d^ | 2.21 ± 0.99 | 4.85 ± 0.12^d^ |
| Salmon^c^ | -19.06 ± 1.07^c^ | 0.19 ± 0.62^d^ | 14.19 ± 0.76 | 3.56 ± 0.09^d^ |

Black bears sampled from Parsnip Plateau and Hart Ranges of the Rocky Mountains, British Columbia, Canada, 1999.

^a^ Vegetation isotopic baseline from common bear forage plants (*n* = 91) sampled in the Besa–Prophet region, BC (1) and from the upper Columbia River basin (*n* = 26), BC (Hobson et al. 2000), and from *Vaccinium* spp (*n* = 7) collected from Gustavus, AK (K. White *unpublished data*).

^b^ Generalized terrestrial meat isotopic baseline averaged from caribou (*Rangifer tarandus*, *n* = 34) and moose (*Alces alces*, *n* = 36) whole hair samples from the Greater Caribou Recovery Area, British Columbia (2), caribou whole hair samples (*n* = 24), caribou red blood cell samples (*n* = 12), moose whole hair samples (*n* = 10), moose hair tip samples (*n* = 6) and moose meat (*n* = 11) from the Besa–Prophet region, BC (1), and from ant (Formicidae) bulk samples (*n* = 4) representing several individuals from the Upper Columbia River Basin, BC (3).

^c^ Generalized chinook (*Oncorhynchus tshawytscha*) isotopic baseline from throughout the Pacific Northwest from (4) (*n* = 51). *Note*: chinook salmon are only available to black bears in the mountain ecoregion.

^d^ Carbon and nitrogen discrimination factors for all food categories were calculated using equations described by Felicetti et al. (5).

**Figure A in S1 File. Relationship between serially diluted extracted black bear (*Ursus americanus*) hair including a 150 pg cortisol spike.** Serially diluted (1:1, 1:2, 1:4, 1:8, 1:16, 1:32) extracted black bear hair after spiking with 150 pg cortisol using the assay standard provided with the commercial Enzyme Immunoassay kit (Salimetrics LLC, State College, PA, USA). Cortisol was added to this black bear sample because the only available sample with adequate hair had a relatively low cortisol level (11 pg/mg) preventing serial dilutions that would remain within the standard curve range.

**Figure B in S1 File. Black bear (*Ursus americanus*) spiking recovery based on five concentrations of cortisol.** Recovery efficiency was 96.01% ± 0.06 SD based on five cortisol concentrations added to black bear hair. Based on Figures 1 and 2 we are confident that there are no interfering steroids in hair samples collected from black bears.

**Table B in S1 File. Cross-reactivity for antibodies used in black bear (*Ursus americanus*) hair cortisol assay.**

| Antibody Specificity | | |
| --- | --- | --- |
| Compound | Spiked Concentration  (ng/mL) | % Cross-reactivity in HS Salivary Cortisol EIA |
| Prednisolone | 100 | 0.568 |
| Prednisone^a^ | 1000 | ND |
| Cortisone | 1000 | 0.13 |
| 11-Deoxycortisol | 500 | 0.156 |
| 21-Deoxycortisol | 1000 | 0.041 |
| 17α-Hydroxyprogesterone^a^ | 1000 | ND |
| Dexamethasone | 1000 | 19.2 |
| Triamcinolone | 1000 | 0.086 |
| Corticosterone | 10,000 | 0.214 |
| Progesterone | 1000 | 0.015 |
| 17β-Estradiol^a^ | 10 | ND |
| DHEA | 10,000 | ND |
| Testosterone | 10,000 | 0.006 |
| Transferrin^a^ | 66,000 | ND |
| Aldosterone^a^ | 10,000 | ND |

^a^ ND is reported for compounds where cross-reactivity was not detected (< 0.0004).

**Table C in S1 File. All data used to evaluate the influence of diet, sex, and social environment on black bear (*Ursus americanus*) hair cortisol concentration.**

| Ecoregion | Sex | δ^13^C  (‰) | δ^15^N  (‰) | Hair  Weight  (mg) | Cortisol  Concentration  (pg/mg) |
| --- | --- | --- | --- | --- | --- |
| Mountains | F | -23.60 | 3.84 | 3.46 | 0.60 |
| Mountains | F | -23.97 | 4.98 | 10.22 | 1.50 |
| Mountains | F | -23.36 | 3.82 | 28.00 | 2.10 |
| Mountains | F | -23.95 | 4.04 | 12.35 | 2.30 |
| Mountains | F | -24.06 | 3.16 | 12.63 | 3.20 |
| Mountains | F | -23.80 | 3.23 | 16.77 | 3.50 |
| Mountains | F | -23.85 | 4.04 | 8.73 | 3.60 |
| Mountains | F | -24.96 | 4.85 | 7.80 | 3.70 |
| Mountains | F | -22.76 | 4.32 | 28.23 | 3.70 |
| Mountains | F | -24.10 | 3.39 | 17.91 | 3.70 |
| Mountains | F | -24.07 | 4.21 | 19.71 | 4.00 |
| Mountains | F | -24.41 | 4.12 | 23.30 | 4.40 |
| Mountains | F | -24.74 | 3.02 | 10.24 | 4.60 |
| Mountains | F | -23.80 | 3.48 | 19.28 | 4.80 |
| Mountains | F | -25.58 | 2.84 | 34.66 | 4.90 |
| Mountains | F | -24.51 | 5.28 | 27.94 | 5.30 |
| Mountains | F | -24.84 | 5.90 | 20.79 | 5.50 |
| Mountains | F | -24.24 | 4.03 | 16.82 | 5.60 |
| Mountains | F | -23.56 | 2.25 | 21.91 | 5.60 |
| Mountains | F | -23.41 | 4.21 | 10.26 | 5.60 |
| Mountains | F | -23.74 | 3.63 | 32.45 | 6.00 |
| Mountains | F | -24.16 | 6.13 | 17.75 | 6.30 |
| Mountains | F | -23.85 | 3.60 | 37.93 | 7.10 |
| Mountains | F | -23.43 | 5.08 | 27.63 | 7.70 |
| Mountains | F | -24.57 | 5.01 | 13.30 | 7.90 |
| Mountains | F | -24.08 | 3.60 | 14.83 | 7.90 |
| Mountains | F | -23.66 | 4.93 | 8.14 | 8.50 |
| Mountains | F | -24.73 | 4.59 | 22.26 | 8.80 |
| Mountains | F | -24.79 | 4.89 | 43.59 | 10.70 |
| Mountains | M | -23.60 | 2.90 | 9.59 | 1.40 |
| Mountains | M | -24.08 | 2.46 | 12.05 | 1.90 |
| Mountains | M | -23.28 | 4.90 | 27.62 | 2.50 |
| Mountains | M | -23.57 | 3.39 | 10.91 | 3.10 |
| Mountains | M | -24.47 | 5.32 | 7.48 | 3.30 |

Table C in S1 File continued

| Mountains | M | -23.76 | 3.70 | 20.80 | 3.60 |
| --- | --- | --- | --- | --- | --- |
| Mountains | M | -23.96 | 4.08 | 10.17 | 3.80 |
| Mountains | M | -23.58 | 4.40 | 21.39 | 3.90 |
| Mountains | M | -24.59 | 3.79 | 12.89 | 4.00 |
| Mountains | M | -23.54 | 2.71 | 7.22 | 4.60 |
| Mountains | M | -23.57 | 3.80 | 18.50 | 4.70 |
| Mountains | M | -23.55 | 3.99 | 13.40 | 5.80 |
| Mountains | M | -24.02 | 3.65 | 12.76 | 6.00 |
| Mountains | M | -24.02 | 3.65 | 12.76 | 6.00 |
| Mountains | M | -23.83 | 3.45 | 17.79 | 6.30 |
| Mountains | M | -23.62 | 2.45 | 13.55 | 7.10 |
| Mountains | M | -23.62 | 2.45 | 13.55 | 7.10 |
| Mountains | M | -23.69 | 2.67 | 7.58 | 7.50 |
| Mountains | M | -24.33 | 4.39 | 2.95 | 7.90 |
| Mountains | M | -24.02 | 2.04 | 12.29 | 8.00 |
| Mountains | M | -25.53 | 4.16 | 17.46 | 8.50 |
| Mountains | M | -24.00 | 3.50 | 5.09 | 10.20 |
| Mountains | M | -23.42 | 4.87 | 7.77 | 10.90 |
| Mountains | M | -24.40 | 3.91 | 10.07 | 11.10 |
| Mountains | M | -24.40 | 3.13 | 4.49 | 11.10 |
| Mountains | M | -23.15 | 5.69 | 21.23 | 12.50 |
| Mountains | M | -24.24 | 4.06 | 1.85 | 16.20 |
| Mountains | M | -24.18 | 3.05 | 14.19 | 18.90 |
| Mountains | M | -24.55 | 4.02 | 1.62 | 20.60 |
| Plateau | F | -22.96 | 3.99 | 16.56 | 1.20 |
| Plateau | F | -23.46 | 3.97 | 17.79 | 1.30 |
| Plateau | F | -23.16 | 3.65 | 19.40 | 1.50 |
| Plateau | F | -23.30 | 5.05 | 10.82 | 2.10 |
| Plateau | F | -23.78 | 3.86 | 24.97 | 2.40 |
| Plateau | F | -23.42 | 3.44 | 26.03 | 2.90 |
| Plateau | F | -23.23 | 5.05 | 15.92 | 3.10 |
| Plateau | F | -23.87 | 3.42 | 20.82 | 3.30 |
| Plateau | F | -23.85 | 4.26 | 3.96 | 3.40 |
| Plateau | F | -23.73 | 3.85 | 15.55 | 3.40 |
| Plateau | F | -22.87 | 5.17 | 14.47 | 3.50 |
| Plateau | F | -23.72 | 5.13 | 21.35 | 3.60 |
| Plateau | F | -23.92 | 4.93 | 7.94 | 3.60 |
| Plateau | F | -23.50 | 4.64 | 19.83 | 4.00 |
| Plateau | F | -22.98 | 4.04 | 7.72 | 4.00 |
| Plateau | F | -23.07 | 4.48 | 13.31 | 4.20 |

Table C in S1 File continued

| Plateau | F | -23.07 | 3.60 | 9.38 | 4.30 |
| --- | --- | --- | --- | --- | --- |
| Plateau | F | -23.24 | 3.67 | 19.56 | 4.70 |
| Plateau | F | -23.90 | 4.10 | 23.13 | 4.80 |
| Plateau | F | -23.55 | 3.40 | 27.38 | 5.10 |
| Plateau | F | -23.28 | 4.77 | 18.18 | 5.30 |
| Plateau | F | -23.99 | 4.15 | 14.61 | 5.50 |
| Plateau | F | -24.04 | 4.25 | 6.55 | 5.70 |
| Plateau | F | -23.10 | 4.25 | 4.88 | 5.90 |
| Plateau | F | -23.05 | 4.36 | 13.37 | 6.30 |
| Plateau | F | -23.58 | 4.40 | 19.72 | 6.50 |
| Plateau | F | -23.60 | 2.75 | 10.52 | 7.00 |
| Plateau | F | -23.82 | 3.63 | 17.51 | 7.10 |
| Plateau | F | -23.34 | 4.54 | 7.73 | 7.20 |
| Plateau | M | -23.88 | 3.63 | 6.99 | 0.50 |
| Plateau | M | -23.80 | 4.41 | 16.99 | 2.10 |
| Plateau | M | -24.16 | 3.75 | 9.64 | 2.30 |
| Plateau | M | -24.23 | 3.55 | 9.80 | 2.50 |
| Plateau | M | -23.97 | 2.91 | 18.72 | 2.90 |
| Plateau | M | -23.39 | 3.45 | 19.58 | 3.00 |
| Plateau | M | -23.71 | 4.38 | 23.24 | 3.10 |
| Plateau | M | -23.71 | 3.67 | 23.65 | 3.10 |
| Plateau | M | -23.93 | 4.23 | 6.70 | 3.50 |
| Plateau | M | -24.10 | 4.92 | 21.44 | 3.70 |
| Plateau | M | -23.24 | 3.71 | 14.40 | 3.70 |
| Plateau | M | -23.76 | 3.58 | 16.06 | 4.70 |
| Plateau | M | -23.50 | 3.55 | 2.34 | 4.70 |
| Plateau | M | -24.38 | 3.47 | 18.87 | 4.80 |
| Plateau | M | -24.35 | 3.44 | 11.39 | 5.30 |
| Plateau | M | -23.70 | 5.24 | 15.15 | 6.20 |
| Plateau | M | -22.89 | 3.78 | 14.31 | 6.30 |
| Plateau | M | -24.03 | 3.63 | 10.11 | 6.60 |
| Plateau | M | -23.32 | 4.81 | 4.51 | 6.90 |
| Plateau | M | -23.62 | 6.19 | 15.01 | 7.60 |
| Plateau | M | -23.17 | 3.78 | 21.69 | 8.00 |
| Plateau | M | -23.55 | 3.91 | 6.57 | 8.10 |
| Plateau | M | -23.95 | 3.81 | 12.24 | 8.20 |
| Plateau | M | -23.80 | 3.85 | 5.38 | 9.90 |
| Plateau | M | -23.97 | 3.62 | 5.26 | 10.60 |
| Plateau | M | -23.65 | 4.17 | 4.09 | 11.80 |
| Plateau | M | -23.28 | 3.16 | 16.83 | 12.40 |

Table C in S1 File continued

| Plateau | M | -23.65 | 4.27 | 3.37 | 17.10 |
| --- | --- | --- | --- | --- | --- |
| Plateau | M | -23.31 | 4.04 | 1.20 | 26.40 |
| Plateau | M | -23.60 | 3.91 | 1.66 | 35.10 |


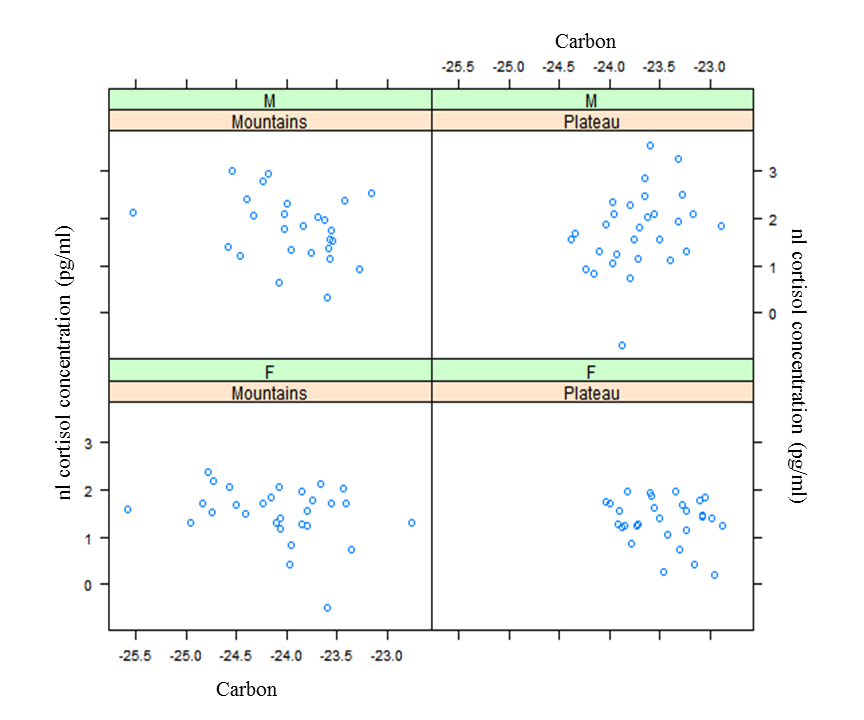


nl cortisol concentration (pg/mg)

nl cortisol concentration (pg/mg)

**Figure C in S1 File. Three-way interaction among carbon, sex, and ecoregion associated with hair cortisol concentration in black bears (*Ursus americanus*).**

## References

1. Milakovic B, Parker KL. Quantifying carnivory by grizzly bears in a multi‐ungulate system. The Journal of Wildlife Management. 2013;77(1):39-47.

2. Steenweg R. Interactions of wolves, mountain caribou and an increased moose-hunting quota-primary-prey management as an approach to caribou recovery: MSc. Thesis. University of Northern British Columbia, Prince George, British Columbia, Canada; 2011.

3. Hobson KA, McLellan BN, Woods JG. Using stable carbon (δ13C) and nitrogen (δ15N) isotopes to infer trophic relationships among black and grizzly bears in the upper Columbia River basin, British Columbia. Canadian Journal of Zoology. 2000;78(8):1332-9.

4. Johnson SP, Schindler DE. Trophic ecology of Pacific salmon (*Oncorhynchus* spp.) in the ocean: a synthesis of stable isotope research. Ecological Research. 2009;24(4):855-63.

5. Felicetti LA, Schwartz CC, Rye RO, Haroldson MA, Gunther KA, Phillips DL, et al. Use of sulfur and nitrogen stable isotopes to determine the importance of whitebark pine nuts to Yellowstone grizzly bears. Canadian Journal of Zoology. 2003;81(5):763-70.
